# High-Volume Hemodiafiltration and Cool Hemodialysis Have a Beneficial Effect on Intradialytic Hemodynamics: A Randomized Cross-Over Trial of Four Intermittent Dialysis Strategies

**DOI:** 10.1016/j.ekir.2022.06.021

**Published:** 2022-07-14

**Authors:** Paul A. Rootjes, Sabrine Chaara, Camiel L.M. de Roij van Zuijdewijn, Menso J. Nubé, Gertrude Wijngaarden, Muriel P.C. Grooteman

**Affiliations:** 1 Department of Nephrology, Amsterdam Cardiovascular Sciences, Amsterdam Universitair Medische Centra, Amsterdam, the Netherlands

**Keywords:** dialysate temperature, hemodiafiltration, hemodialysis, hemodynamic stability, intradialytic hypotension, randomized cross-over trial

## Abstract

**Introduction:**

Compared to standard hemodialysis (S-HD), postdilution hemodiafiltration (HDF) has been associated with improved survival.

**Methods:**

To assess whether intradialytic hemodynamics may play a role in this respect, 40 chronic dialysis patients were cross-over randomized to S-HD (dialysate temperature [Td] 36.5 °C), cooled HD (C-HD; Td 35.5 °C), and HDF (low-volume [LV-HDF)] and high-volume [HV-HDF], both Td 36.5 °C, convection volume 15 liters, and at least 23 liters per session, respectively), each for 2 weeks. Blood pressure (BP) was measured every 15 minutes. The primary endpoint was the number of intradialytic hypotensive (IDH) episodes per session. IDH was defined as systolic BP (SBP) less than 90 mmHg for predialysis SBP less than 160 mmHg and less than 100 mmHg for predialysis SBP greater than or equal to 160 mmHg, independent of symptoms and interventions. A *post hoc* analysis on early-onset IDH was performed as well. Secondary endpoints included intradialytic courses of SBP, diastolic BP (DBP) and mean arterial pressure (MAP).

**Results:**

During S-HD, IDH occurred 0.68 episodes per session, which was 3.2 and 2.5 times higher than during C-HD (0.21 per session, *P* < 0.0005) and HV-HDF (0.27 per session, *P* < 0.0005), respectively. Whereas the latter 2 strategies showed similar frequencies, HV-HDF differed significantly from LV-HDF (*P* = 0.02). A comparable trend was observed for early-onset IDH: S-HD (0.32 per session), C-HD (0.07 per session, *P* < 0.0005) and HV-HDF (0.10 per session, *P* = 0.001). SBP, DBP, and MAP declined during S-HD (−6.8, −5.2, −5.2 mmHg per session; *P* = 0.004, *P* < 0.0005, *P* = 0.002 respectively), which was markedly different from C-HD (*P* < 0.01).

**Conclusion:**

Though C-HD and HV-HDF showed the lowest IDH frequency and the best intradialytic hemodynamic stability, all parameters were most disrupted in S-HD. Therefore, the survival benefit of HV-HDF over S-HD may be partly caused by a more beneficial intradialytic BP profile.

## Graphical abstract

**Figure:**
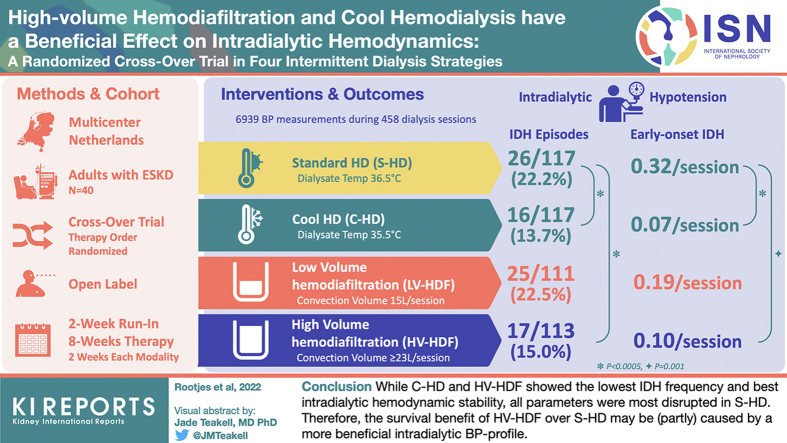

Despite major improvements in patient care and dialysis equipment over the past decades, chronic hemodialysis (HD) patients still have an unacceptably high mortality rate, which is much higher than in the general population.^1^ In this respect, fatal cardiovascular (CV) disease accounts for the vast majority of deaths.^2^ A high prevalence of traditional risk factors, such as hypertension, diabetes mellitus, and dyslipidemia,^3^, ^4^, ^5^, ^6^ coupled with risk factors that are specific for chronic kidney disease (CKD), such as derangements of the calcium-phosphate metabolism, fluid overload, anemia, inflammation, and oxidative stress,^5^^,^^7^^,^^8^ is responsible for the high risk of CV disease.^2^^,^^5^^,^^9^, ^10^, ^11^ Furthermore, side-effects of the HD procedure itself, such as the bio-incompatibility of the extra-corporeal circuit,^12^ fluctuations in acid-base equilibrium and IDH, may contribute to the poor clinical outcome as well.

In patients with end-stage kidney disease without residual diuresis, the fluid that accumulates during the interdialytic interval must be removed during the next dialysis. Yet, when the ultrafiltration rate exceeds the plasma refill rate, blood volume will decline. Combined with insufficient compensatory CV responses to maintain an adequate BP, IDH can occur.^13^, ^14^, ^15^ Besides subjective discomfort,^16^^,^^17^ IDH also induces repetitive ischemia in vital organs, including the heart, brain, kidney, and gut.^14^^,^^18^, ^19^, ^20^ Depending on the definition used, IDH occurs in 10% to 30% of the dialysis sessions.^21^ Interestingly, a large retrospective study comparing 8 different IDH definitions, revealed that a SBP less than 90 mmHg or less than 100 mmHg (with a predialysis SBP less than 160 mmHg or more than or equal to 160 mmHg, respectively) was most strongly associated with mortality.^22^ In addition, it was recently demonstrated that especially early-onset IDH (IDH ≤120 minutes after the start of HD) is associated with a poor prognosis.^23^ Notably, both HD with a low dialysate temperature ([Td]; cool HD [C-HD]) and online postdilution hemodiafiltration (HDF), which combines diffusive with convective transport, may lower the frequency of IDH in comparison with “standard” HD (S-HD).^24^, ^25^, ^26^, ^27^, ^28^ Yet, well executed studies comparing the incidence of IDH in detail between S-HD, C-HD and HDF are lacking.

A previous meta-analysis on the individual patient data of 4 randomized controlled trials^29^ indicated that HDF is associated with a superior overall and CV survival, if compared to S-HD. The largest benefit was observed in patients who achieved the highest convection volume (high-volume [HV]-HDF).^29^ Currently, however, the mechanism behind this effect is unknown. Becuase IDH, as above-mentioned, has been associated with a poor clinical outcome and HDF may reduce its incidence, it is conceivable that the superior survival of HDF over HD may be due to more stable intradialytic hemodynamics.

Therefore, in the present study, we compared the following: (i) the number of (early) IDH episodes per dialysis session and (ii) the intradialytic courses of SBP, DBP, and MAP between S-HD, C-HD, LV-HDF, and HV-HDF.

## Methods

### Study Design

This study (ClinicalTrials.gov identifier NCT03249532) is an open-label, multicenter, randomized cross-over trial, in prevalent dialysis patients. The methods have been described in detail elsewhere.^30^ In summary, the patients were subjected to the following 4 extracorporeal renal replacement therapies in a random order: (i) S-HD (Td 36.5°C), (ii) C-HD (Td 35.5°C), (iii) HDF (Td 36.5°C) with a target convection volume of 15 liter per session (LV-HDF), and (iv) HDF (Td 36.5°C) with a target convection volume of at least 23 liters per session (HV-HDF). Total study duration was 10 weeks, divided into a two-week run-in period and an eight-week experimental phase (2 weeks per modality). After enrollment, patients were randomly assigned to a certain treatment order. Due to the nature of the intervention, it was impossible to conceal the type of extracorporeal renal replacement therapies. The study was conducted in accordance with the Declaration of Helsinki and Good Clinical Practice guidelines and approved by the Medical Ethical committee of VU University medical center (METC VUmc: 2017.581/NL61210.029.17). Written informed consent was obtained from all patients prior to enrollment.

### Sample Size Calculation

A power calculation showed that a total of 40 patients with complete follow-up would be sufficient to detect a 40% lower risk (relative risk of 0.60, α = 0.05, β = 0.80) of the primary endpoint. The power calculation applied was designed for cross-over studies.^31^ Accounting for a loss-to follow-up of 10%, we aimed at including 44 patients.

### Study Population

From July 2018 to February 2021, patients were recruited from 3 dialysis centers in the Netherlands described as follows: 1 out-of-hospital facility (Niercentrum aan de Amstel, Amstelveen), 1 facility within an academic hospital (Amsterdam UMC, location VU University medical center, Amsterdam), and 1 facility within a community-based hospital (Sint Antonius Ziekenhuis, Nieuwegein). Inclusion criteria were as follows: (i) treatment with HD or HDF 3 times per week during 4 hours for at least 2 months, (ii) ability to understand the study procedure, (iii) willingness to provide informed consent, (iv) dialysis single-pool Kt/V for urea greater than or equal to 1.2, (v) blood flow greater than or equal to 350 ml/min during the run-in phase, and (vi) most recent dialysis access recirculation less than 10%. Exclusion criteria were as follows: (i) age less than 18 years, (ii) life expectancy less than 3 months, (iii) participation in another clinical intervention trial, and (iv) severe noncompliance to the dialysis procedure and accompanying prescriptions.

### Dialysis Prescription and Equipment

All treatments were performed with Xevonta 23 high-flux dialyzers (B. Braun Avitum AG, Melsungen, Germany) and treatment times were fixed at 4 hours per session. HDF was performed online in the postdilution mode. Extracorporeal blood flow rate was targeted at 350 ml/min to 400 ml/min, and filtration fraction (blood flow rate/convection flow rate) at 25% to 30%. All dialysis treatments were performed on Dialog iQ dialysis machines, including the captive lines Diastream (both B. Braun Avitum AG, Melsungen, Germany). Ultrapure dialysis fluids (less than 0.1 colony forming units/ml, less than 0.03 endotoxin units/ml) were mixed using Sol-Cart Bicarbonate cartridge and acidic dialysate. Substitution fluid was prepared from the dialysis fluid by an additional step of ultrafiltration with a dialysis fluid filter (Diacap Ultra, B. Braun Avitum AG, Melsungen, Germany), before infusing into the blood. For a given patient, treatment settings were kept similar in all treatment modalities. All patients received their usual dose of low molecular weight heparin anticoagulation (i.e., nadroparin or dalteparin). Routine patient care was performed according to national and international quality of care guidelines.^32^^,^^33^

### Primary and Secondary Endpoints

The primary endpoint was the average number of IDH episodes per dialysis session. IDH was defined as a SBP less than 90 mmHg for a predialysis SBP less than 160 mmHg, or SBP less than 100 mmHg for a predialysis SBP greater than or equal to 160 mmHg, independent of symptoms and interventions.^22^ A *post hoc* analysis on the incidence of early-onset IDH (≤120 min after the start of dialysis) was performed as well.^23^ Secondary endpoints included the intradialytic courses (rate of change) of SBP, DBP and MAP.

### Data Collection

#### Clinical Measurements

At baseline, information on demographics, history of CV disease, primary renal diagnosis, comorbidity, medical history and medications were obtained. Body weight and interdialytic weight gain were assessed before dialysis. Data on the type of vascular access, access flow, anticoagulation type, needle size and type, blood pump speed, dialysis machine, and dialyzer were documented as well. For HDF, the achieved convection volume, calculated as the sum of intradialytic weight loss (net ultrafiltration) and substitution volume in liters per session, was noted. Body weight was recorded after each dialysis procedure. Body temperature (T_b_) was measured before and after each dialysis session with a tympanic thermometer (Genius 2 Tympanic Thermometer, Covidien, Mansfield, USA).

#### Hemodynamic Monitoring

During the 3 treatment sessions in the second week of each modality, BP was recorded both at the start and every 15 minutes thereafter using an automated manometric cuff device connected to the dialysis machine (Adimea, automatic BP monitor, B. Braun Avitum AG, Melsungen). This device provides measurements of SBP, DBP and heart rate. MAP was calculated with the formula: MAP = ([SBP + 2∗DBP]/3).

#### Statistical Analyses

Baseline characteristics were summarized as mean ± SD for normally distributed continuous variables, median and interquartile range for nonGaussian distributed continuous variables and counts with percentages for categorical variables. A dichotomous variable was created to assess the occurrence of IDH during a single dialysis session. Furthermore, the average number of IDH episodes per dialysis session of each dialysis modality (episodes per session) was calculated. Next, the Kolmogorov-Smirnov test was used to determine whether the number of IDH episodes followed a Poisson distribution. As the data appeared to be overdispersed (*P* < 0.0005), we used negative binomial regression analysis to evaluate our primary endpoint. Various reference categories were used to determine potential differences between all modalities. Hereafter, the average number of IDH episodes per session was subdivided into early- and late-onset (respectively ≤120 and >120 minutes after the start of dialysis) IDH. As these parameters appeared to be overdispersed as well (*P* < 0.0005), negative binomial regression analyses were used again. Lastly, to analyze the intradialytic courses of SBP, DBP and MAP and to assess whether the courses differed between the 4 treatment modalities, we used linear mixed models with an interaction term between time and modality and calculated the intradialytic rate of change per hour. A *P*-value for interaction less than 0.1 was considered potentially relevant. Stratified models were fitted subsequently. For all linear mixed models, a random slope, random intercept or both a random slope and a random intercept were used, according to the lowest Aikaike’s Information Criterion. Analyses were performed using the statistical software package SPSS version 26.0 (IBM Inc., IL, USA). In general, a *P* ≤ 0.05 was considered statistically significant. However, to adjust for multiple testing and thus minimize the occurrence of a type I statistical error, correction according to the Holm-Bonferroni method was applied.^34^

#### Sensitivity Analyses

To increase the robustness of our findings, complete case analyses of all previously mentioned analyses were performed. These included only participants who were exposed to all treatment modalities and had less than 25% missing BP measurements per session.

## Results

### Patient Characteristics

**Figure 1:**
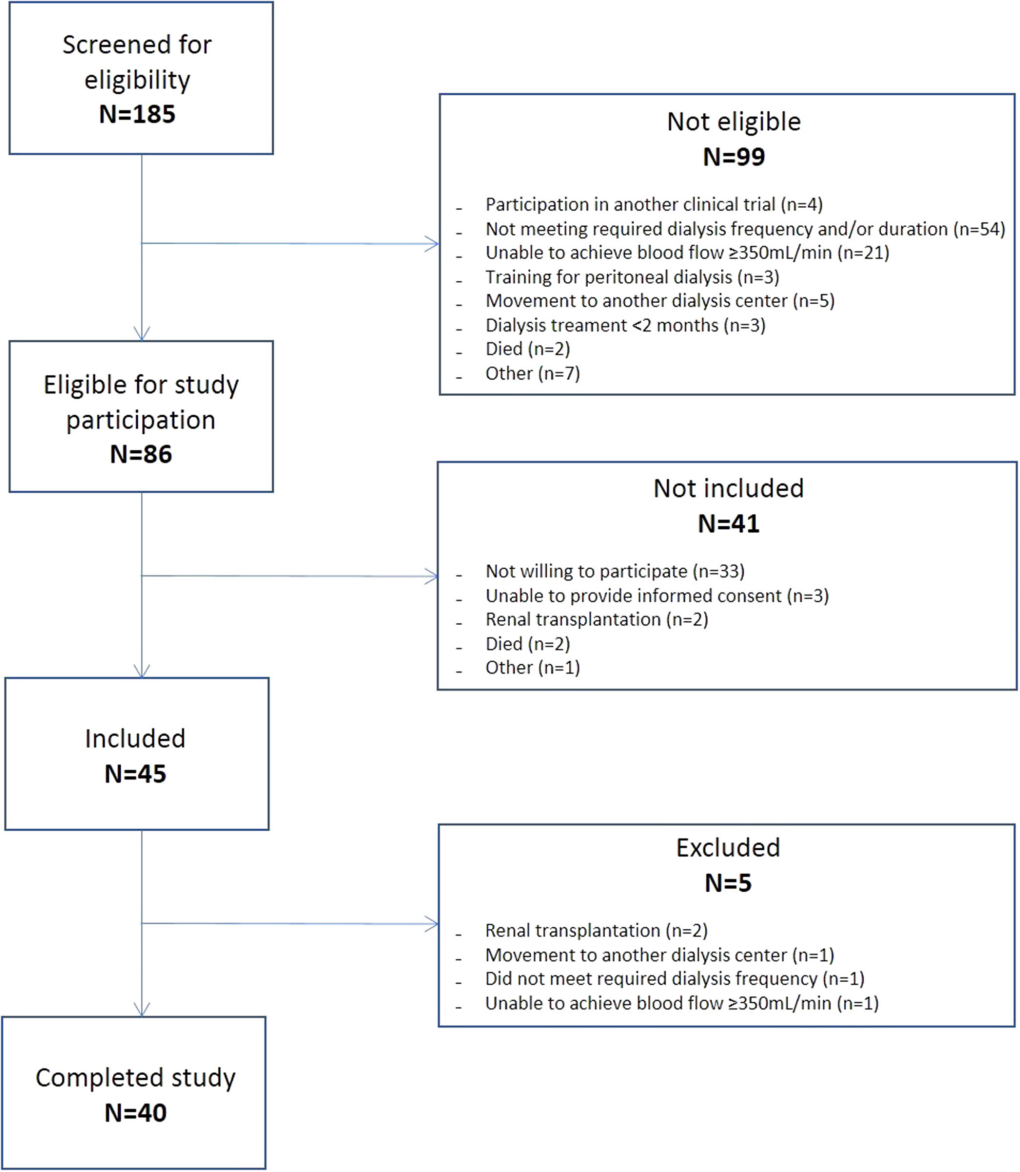
Study flowchart.

As shown in Figure 1, 45 patients were included. Before randomization, 5 patients dropped out due to renal transplantation (*n* = 2), movement to another dialysis facility (*n* = 1), not meeting the required dialysis treatment frequency (*n* = 1), and inability (due to access problems) to achieve a blood flow of at least 350 ml/min (*n* = 1). Baseline demographic and clinical characteristics, laboratory data, medication and treatment-related parameters are summarized in Table 1. Most patients were males (75%) and mean age was 69.7 ± 13.5 years. Median dialysis vintage was 3.0 years (interquartile range 1.0–5.8).

**Table 1:** Baseline characteristics of study participants (*N* = 40)

Characteristics	*N* = 40
Demographics	
Sex (male)	30 (75%)
Age (yrs)	69.7 ± 13.5
Race: Caucasian/African/Asian	28/10/2 (70%/25%/5%)
Clinical characteristics	
BMI (kg/m^2^)	26.7 ± 4.2
Smoking status: Non/former/current	14/18/8 (35%/45%/20%)
SBP, predialysis (mmHg)	145 ± 23
DBP, predialysis (mmHg)	81 ± 13
Residual kidney function^a^	24 (60%)
Residual kidney function (ml/min)^b^	1.9 (1.0–2.5)
Medical history	
Dialysis modality: HDF	17 (42%)
Dialysis vintage (yrs)	3.0 (1.0–5.8)
History of kidney transplantation	3 (8%)
Primary cause of ESRD	
Glomerulonephritis	10 (25%)
Renal vascular disease	9 (23%)
Diabetic nephropathy	15 (38%)
Cystic kidney disease	1 (3%)
Other/Unknown	4 (10%)/1 (3%)
Diabetes mellitus	19 (48%)
Hypertension	28 (70%)
History of CVD	29 (73%)
Medication	
ACE-I/ARB	10 (25%)
Beta blockers	25 (63%)
Calcium antagonists	10 (25%)
Diuretics	11 (28%)
ESA	32 (80%)
Laboratory data	
Hemoglobin (mmol/l)	7.1 ± 0.7
Creatinine (μmol/l)	865 ± 229
Sodium (mmol/l)	138 ± 4
Potassium (mmol/l)	5.1 ± 0.6
Phosphate (mmol/l)	1.6 ± 0.5
Albumin (g/l)	38.6 ± 4.5
PTH (pmol/l)	28.2 (15.1–48.3)
Dialysis parameters	
Vascular access: AVF/Graft/CVC	32/4/4 (80%/20%/20%)

ACE-I, angiotensin-converting enzyme inhibitor; ARB, angiotensin receptor blocker; AVF, arteriovenous fistula; BMI, body mass index; CVC, central venous catheter; CVD, cardiovascular disease; DBP, diastolic blood pressure; ESA, erythropoiesis-stimulating agent; ESRD, end-stage renal disease; HD, hemodialysis; HDF, hemodiafiltration; PTH, parathyroid hormone; SBP, systolic blood pressure.

Values are number (n) (%) for categorical variables, and mean ± SD or median (interquartile range 25%–75%) for continuous variables. Laboratory data are predialytic values.

a Residual diuresis >100 ml/24 h.

b In patients with diuresis >100 ml/24 h.

### Missing Data

Of the 40 patients who finished the study, 2 were not exposed to HDF due to technical failure but completed S-HD and C-HD. Two patients withdrew their consent after completing 75% and 50% of the study. The total amount of missing BP values was 126 (6.3%) for S-HD, 81 (4.1%) for C-HD, 91 (4.8%) for LV-HDF, and 97 (5.1%) for HV-HDF.

### Treatment Characteristics

Dialysis characteristics are shown in Table 2. Mean blood flow was 339 ± 33 ml/min for S-HD, 332 ± 41 for C-HD, 339 ± 36 for LV-HDF, and 347 ± 27 for HV-HDF. Mean total ultrafiltration volume was 2.4 ± 0.7 l per session for C-HD and 2.3 ± 0.7 for the other modalities. Mean total convection volume was 15.1 ± 1.3 liters per session for LV-HDF and 22.6 ± 1.1 liters per session for HV-HDF. T_b_ appeared to increase similarly during S-HD, LV-HDF and HV-HDF. During C-HD, however, T_b_ remained stable (Supplementary Table S1).

**Table 2:** Dialysis characteristics

Modality	Blood flow (ml/min)	Dialysate flow (ml/min)	Total UF (l/session)	Total convection volume (l/session)
S-HD	339 ± 33	505 ± 11	2.3 ± 0.7	N/A
C-HD	332 ± 41	505 ± 13	2.4 ± 0.7	N/A
LV-HDF	339 ± 36	590 ± 19	2.3 ± 0.6	15.1 ± 1.3
HV-HDF	347 ± 27	594 ± 18	2.3 ± 0.7	22.6 ± 1.1

C-HD, cool hemodialysis; LV-HDF, low-volume hemodialysis; HV-HDF, high-volume hemodialysis; S-HD, standard hemodialysis; N/A= not applicable; UF, ultrafiltration.

Mean ± SD for blood flow, dialysate flow, total ultrafiltration volume; and total convection volume.

### Hemodynamic Stability

#### Intradialytic Hypotension

**Figure 2:**
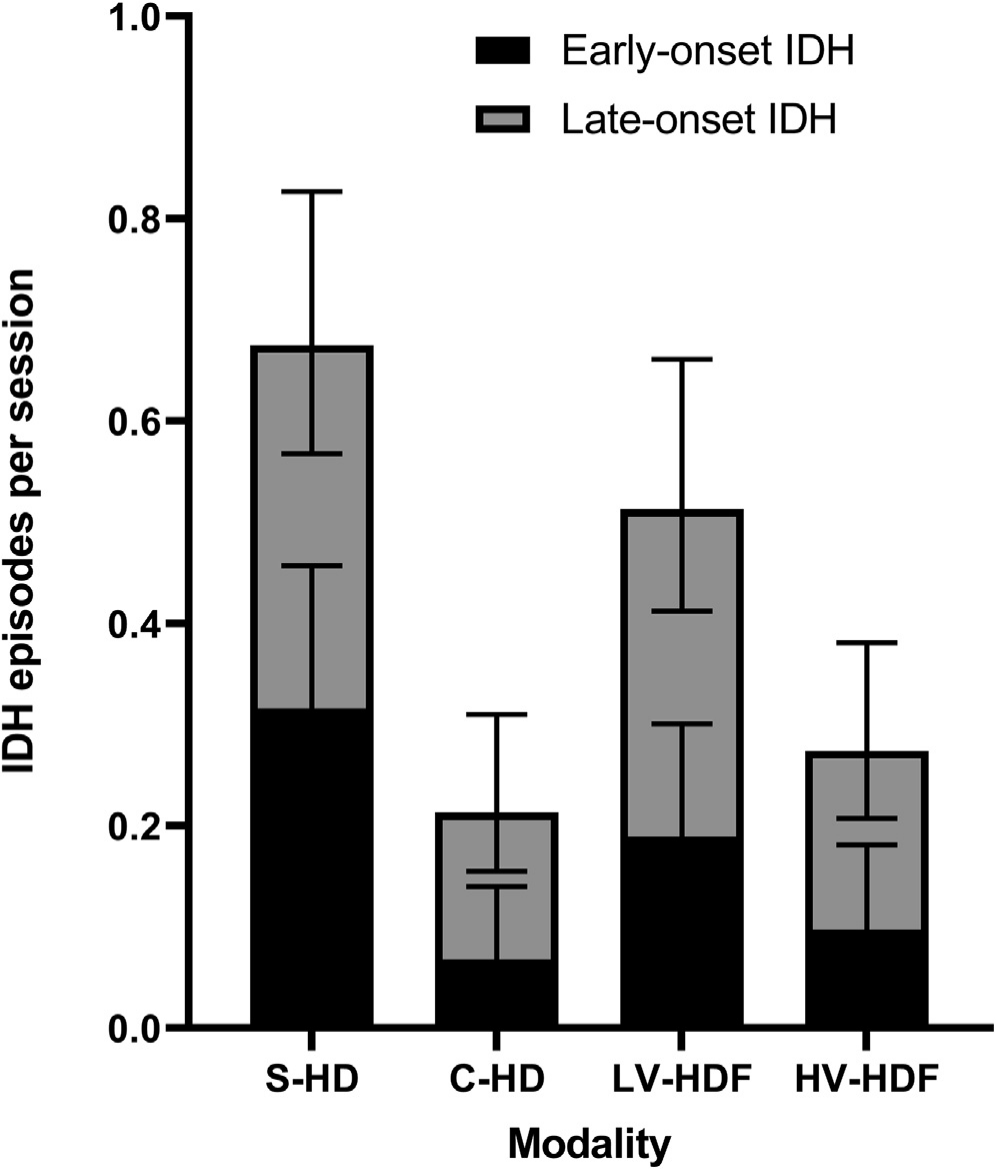
Average number of IDH (definition see text) episodes per session of each dialysis modality with 95% confidence interval, subdivided into early- and late-onset IDH (resp. ≤120 minutes and >120 minutes after the start of dialysis). For the corresponding *P*-values, see Table 3. C-HD, cool hemodialysis; IDH, intradialytic hypotensive; LV and HV-HDF: low-volume and high-volume hemodiafiltration; S-HD, standard hemodialysis.

Altogether, 6939 BP measurements were performed during 458 dialysis sessions. IDH was observed in 26 of 117 (22.2%) sessions in S-HD, 16 of 117 (13.7%) in C-HD, 25 of 111 (22.5%) in LV-HDF and 17 of 113 (15.0%) in HV-HDF. As shown in Figure 2 and Table 3, the average number of IDH episodes per dialysis modality was 0.68 per session in S-HD, 0.21 per session in C-HD, 0.51 per session in LV-HDF and 0.27 per session in HV-HDF. Whereas the differences between S-HD, and both C-HD and HV-HDF were highly significant (*P* < 0.0005), C-HD and HV-HDF were comparable in this respect (*P* = 0.40). Interestingly, the number of IDH episodes per session differed significantly between LV-HDF and HV-HDF (*P* = 0.02). Sensitivity analysis yielded similar results (Supplementary Table S2).

**Table 3:** Average number of IDH episodes (overall and early) per modality

Modality	Total IDH episodes per session	*P*-value^a^	Early IDH episodes per session	*P*-value^a^
S-HD	0.68	Ref	0.32	Ref
C-HD	0.21	<0.0005^b^	0.07	<0.0005^b^
LV-HDF	0.51	0.21	0.19	0.09
HV-HDF	0.27	<0.0005^b^	0.10	0.001^b^

C-HD, cool hemodialysis; IDH, intradialytic hypotensive; LV-HDF, low-volume hemodialysis; HV-HDF, high-volume hemodialysis; S-HD, standard hemodialysis.

Average number of total IDH episodes and early-onset IDH (≤ 120 minutes after start of dialysis) episodes per session during S-HD, C-HD, LV-HDF and HV-HDF.

a *P* for difference in number of intradialytic hypotensive episodes.

b Statistically significant after correction for multiple testing by the Holm-Bonferroni method.

### Early-Onset Intradialytic Hypotension

The average numbers of early-onset IDH episodes are shown in Figure 2 and Table 3. As shown in the graph (Figure 2), both early-onset and late-onset IDH occurred most frequently in S-HD. The number of early-onset IDH episodes differed significantly between S-HD (0.32/session) and both C-HD (0.07/session; *P* < 0.0005) and HV-HDF (0.10/session; *P* = 0.001). Differences were neither found between C-HD and HV-HDF (*P* = 0.47), nor between HV-HDF and LV-HDF (*P* = 0.09). Sensitivity analysis yielded similar results (Supplementary Table S3).

### Intradialytic Courses of BP Parameters

**Figure 3:**
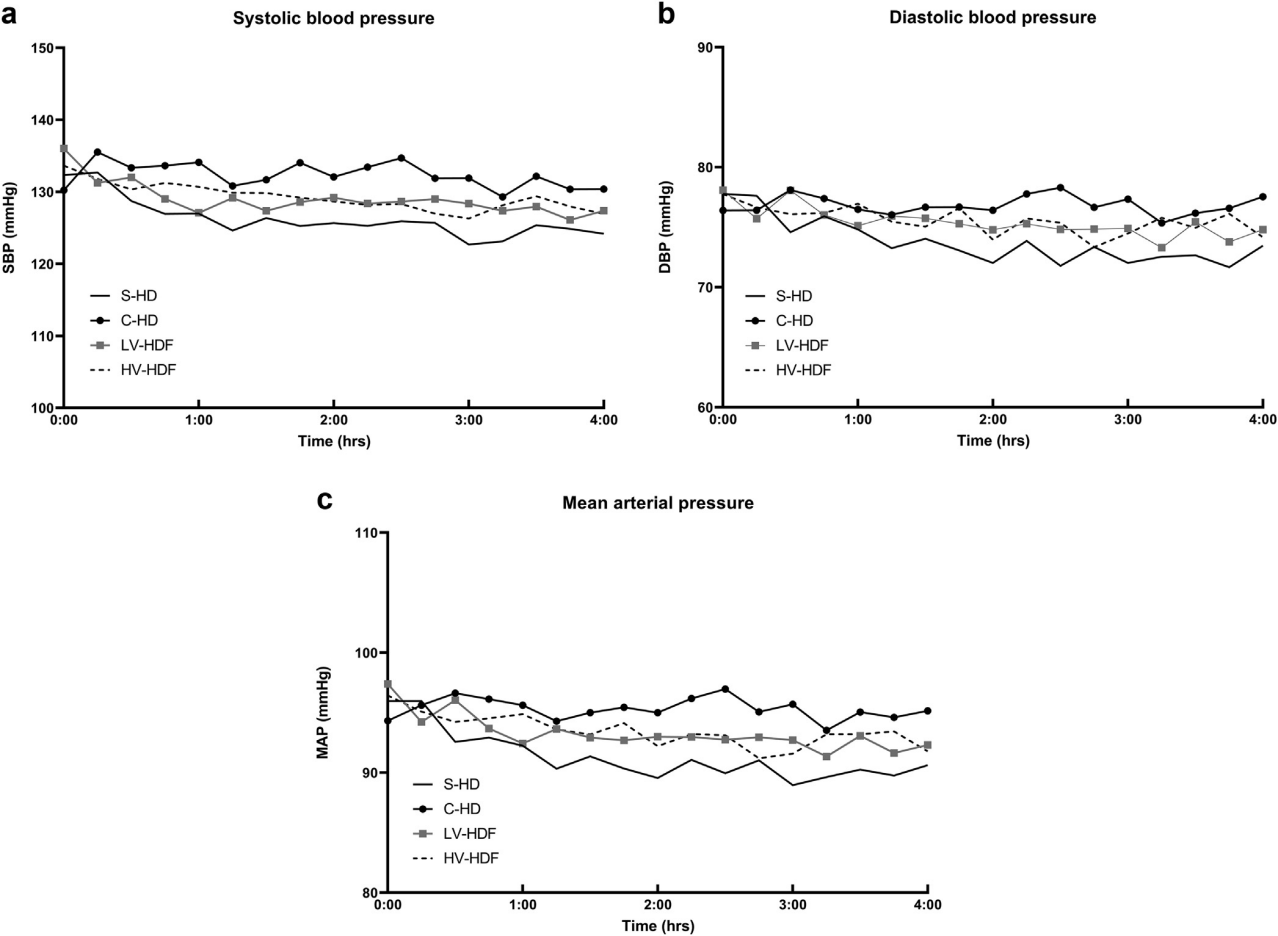
(A) Intradialytic courses of mean SBP; (B) mean diastolic BP; (C) mean arterial pressure during S-HD, C-HD, LV-HDF and HV-HDF. SBP, DBP and mean arterial pressure all declined significantly during S-HD (*P* = 0.004, *P* < 0.0005, *P* = 0.002 respectively). C-HD, cool hemodialysis; LV and HV-HDF, low-volume and high-volume hemodiafiltration; S-HD, standard hemodialysis; SBP, systolic blood pressure.

Although the courses of both SBP, DBP and MAP appeared to decline during all modalities, after correction for multiple testing, the intradialytic drops were only significant in the case of S-HD (−6.8, −5.2, −5.2 mmHg/session; *P* for declines: *P =* 0.004, *P* < 0.0005, and *P =* 0.002 respectively), which differed markedly from C-HD (*P* for interaction: *P* = 0.006, *P* < 0.0005 and *P* < 0.0005 respectively; Figure 3 and Table 4).

**Table 4:** Mean intradialytic rate of change of blood pressure

Blood pressure	*P* for interaction	Change (mmHg) per hour (95% CI)	*P* for change per hour
SBP			
S-HD	Ref	−1.7 (−2.8 to −0.6)	0.004^a^
C-HD	0.006^a^	−0.7 (−2.0 to 0.7)	0.29
LV-HDF	0.29	−1.3 (−2.4 to −0.1)	0.04
HV-HDF	0.55	−1.4 (−2.5 to −0.6)	0.01
DBP			
S-HD	Ref	−1.3 (−2.0 to −0.6)	<0.0005^a^
C-HD	<0.0005^a^	0.1 (−0.7 to 0.8)	0.91
LV-HDF	0.09	−0.8 (−1.6 to −0.1)	0.04
HV-HDF	0.06	−0.8 (−1.4 to −0.1)	0.02
MAP			
S-HD	Ref	−1.3 (−2.2 to 0.5)	0.002^a^
C-HD	<0.0005^a^	−0.2 (−1.1 to −0.7)	0.69
LV-HDF	0.15	−0.9 (−1.7 to −0.1)	0.04
HV-HDF	0.23	−1.0 (−1.7 to −0.2)	0.01

CI, confidence interval; C-HD, cool hemodialysis; DBP, diastolic blood pressure; LV-HDF, low-volume hemodialysis; HV-HDF, high-volume hemodialysis; MAP, mean arterial pressure; S-HD, standard hemodialysis; SBP, systolic blood pressure.

Mean intradialytic rate of change per hour for SBP, DBP and MAP in mmHg with 95% CI and *P*-values for stratified linear mixed models.

a Statistically significant after correction for multiple testing by the Holm-Bonferroni method.

## Discussion

The present analysis clearly shows that S-HD is associated with the highest IDH incidence per session, and both C-HD and HV-HDF with the lowest. To our knowledge, this is the first randomized cross-over study comparing hemodynamic stability during 4 frequently used intermittent dialysis modalities. In comparison with S-HD, especially HV-HDF has been associated with a beneficial effect on survival^29^ and C-HD in particular with a stabilizing effect on intradialytic BP.^26^^,^^35^^,^^36^ Therefore, we were especially interested in whether the intradialytic hemodynamics differ between S-HD and HV-HDF, and whether C-HD differs from HV-HDF in these respects. Finally, to assess the influence of the convection volume on these parameters, we compared LV-HDF with HV-HDF.

An important aspect of our study is the fact that the IDH definition used showed the strongest association with mortality out of 8 different IDH definitions.^22^ In addition, the discrimination between IDH with and without symptoms and/or interventions, as used in official guidelines,^37^ was not substantiated by that study.^22^ Therefore, we analyzed all IDH episodes, irrespective of concurrent intradialytic symptomatology and/or subsequent interventions. Since it was recently demonstrated that especially early-onset IDH is associated with an increased mortality risk,^23^ a *post hoc* analysis on this parameter was performed as well. Altogether, our findings largely confirm prior studies, which also reported a lower incidence of IDH during both HV-HDF and C-HD than during S-HD,^24^^,^^26^, ^27^, ^28^^,^^38^ but a similar incidence during HV-HDF and C-HD.^24^^,^^39^ Yet, and in contrast to the current analysis, most of these studies were limited by less frequent BP measurements (twice/hour vs. 4 times/hour) and/or the absence of a cross-over design. It should be acknowledged, however, that IDH has not only been associated with mortality, but also with morbidity, most likely due to chronic repetitive perfusion deficits leading to tissue ischemia and organ dysfunction.^40^^,^^41^ In fact, several manifestations of organ damage have been described, including myocardial stunning,^42^ brain atrophy and dementia,^43^, ^44^, ^45^ loss of residual kidney function,^46^ and mesenteric ischemia.^47^

Considering the secondary endpoints, SBP, DBP, and MAP all declined significantly during S-HD and remained relatively stable during the other dialysis modalities. Whereas marked differences existed between S-HD and HV-HDF, the intradialytic hemodynamic patterns during C-HD and HDF (HV as well as LV) were similar. Since MAP is a valid indicator of tissue perfusion,^48^, ^49^, ^50^, ^51^, ^52^, ^53^ it appears that S-HD is the worst dialysis strategy in this respect for the long-term treatment of patients with end-stage kidney disease.

As for the pathophysiological background, our results may support the idea that thermal effects are important BP-stabilizing factors.^24^^,^^26^^,^^39^^,^^54^ Due to the loss of thermal energy within the extra-corporeal circuit^24^ during C-HD and HDF and the subsequent cooling effect on central T_b_,^39^ peripheral micro-vessels constrict in an attempt to reduce heat loss and keep T_b_ within limits. Since DBP is particularly determined by total peripheral resistance,^55^ and cutaneous vasoconstriction is a functional adaptation to a decline in T_b_, DBP may remain relatively unchanged during HV-HDF and C-HD. In S-HD (Td 36.5°C), however, heat loss is restricted and T_b_ may remain constant or even rise.^39^ As a result, the peripheral microcirculation may dilate and, consequently, induce a decline in DBP and MAP. Due to a decrease in venous return and a dysfunction of the baroreflex as observed in many CKD patients,^51^, ^52^, ^53^ cardiac output and SBP may decline as well. Since the replacement fluid (T 36.5°C), which is administered in HDF, cools down in the extra-corporeal circuit which is exposed to room temperature, any increase in substitution volume, as in HV-HDF, may lower T_b_ further^39^ and, hence, the incidence of IDH.^54^ Indeed, in our study IDH was significantly less often observed during HV-HDF than during LV-HDF. Yet, since Tb increased similarly in both HDF modalities and S-HD, besides thermal influences^39^ other mechanisms, such as direct intradialytic cardiac protective effects and/or reductions in oxidative stress,^56^, ^57^, ^58^, ^59^ may be involved as well.

Important strengths of this study are its randomized cross-over design, its adequate power, and meticulous data collection. Because patients serve as their own controls in a cross-over setting, between-subject variability is eliminated. Another strength is the frequency of BP measurements, which was every 15 minutes, compared to every 30 minutes or more in most previous studies. Lastly, sensitivity analyses increase the robustness of our findings. Nonetheless, some limitations need to be acknowledged as well. A wash-out period of one week may be too short to exclude carry-over effects. Yet, since data on the duration of modality-induced BP changes are not available, one week was considered sufficient. In addition, since diabetes mellitus is more prevalent in our study than in the European dialysis population (48% vs. 31%),^60^ our results may not be generalizable to other populations. Furthermore, the results of the HV-HDF group may be an underestimation of the true effect, since in some patients the target convection volume of at least 23 liters per session could not be achieved. As for the mechanism(s) behind the dampening effect of both HV-HDF and C-HD on IDH, in retrospect it is regrettable that we could not ascertain which mechanism(s) is or are responsible for the observed effects. Yet, because the objective of the present study was completely different, future studies aimed to investigate the causative background of IDH and its prevention, can solve this issue. Finally, since patients were instructed to continue all medication, including beta blockers which may be cleared differently during high-flux HD and HDF, a dissimilar effect on hemodynamic stability during treatment cannot be ruled out.^61^

To summarize, our study first reveals that IDH, which has been associated with an unfavourable outcome, occurs least frequently during both C-HD and HV-HDF. Second, it appears that both SBP, DBP, and MAP decline significantly during S-HD, and remain relatively stable during the other modalities. Since the intradialytic hemodynamics are most disrupted during S-HD, it is conceivable that the survival benefit of HV-HDF over S-HD is at least partly due to a better preserved intradialytic hemodynamic profile. Yet, which dialysis mode becomes first choice in daily practice, will also depend on future survival studies, environmental impact, patient preferences, and patient quality of life.

## Disclosure

MJN, MPCG and PAR report unrestricted grant support for the present study from Niercentrum aan de Amstel, Elyse Klinieken, the Netherlands, and B. Braun Avitum AG, Melsungen, Germany. All the other authors declared no competing interests. The unrestricted grants were provided to the Amsterdam UMC, location Vrije Universiteit Amsterdam. Niercentrum aan de Amstel is owned by both Elyse Klinieken and Amsterdam UMC, location Vrije Universiteit Amsterdam, the Netherlands. There was no involvement in the collection, analysis and interpretation of data, or in the reporting of the results. Moreover, members of the study team were not employed by B. Braun Avitum AG or Niercentrum aan de Amstel.

## Supplementary Material

Supplementary File (PDF)

**Table S1.** Body temperature before and after dialysis

**Table S2.** Sensitivity analysis average number of IDH episodes

**Table S3.** Sensitivity analysis average number of early IDH episodes
